# Driving fibrosis in neuromuscular diseases: Role and regulation of Connective tissue growth factor (CCN2/CTGF)

**DOI:** 10.1016/j.mbplus.2021.100059

**Published:** 2021-03-06

**Authors:** Daniela L. Rebolledo, Kenneth E. Lipson, Enrique Brandan

**Affiliations:** a Centro de Envejecimiento y Regeneración, CARE Chile UC, Chile; b Departamento de Biología Celular y Molecular, Facultad de Ciencias Biológicas, Pontificia Universidad Católica de Chile, Chile; c FibroGen Inc., San Francisco, CA, USA; d Centro de Excelencia en Biomedicina de Magallanes (CEBIMA), Punta Arenas, Chile; e Fundación Ciencia y Vida, Santiago, Chile

**Keywords:** ALS, Amyotrophic Lateral Sclerosis, CCN2/CTGF, connective tissue growth factor, DMD, Duchenne muscular dystrophy, ECM, extracellular matrix, LPA, lysophosphatidic acid, CCN2/CTGF, Skeletal muscle, Fibrosis, FG-3019, Neuromuscular diseases

## Abstract

Connective tissue growth factor or cellular communication network 2 (CCN2/CTGF) is a matricellular protein member of the CCN family involved in several crucial biological processes. In skeletal muscle, CCN2/CTGF abundance is elevated in human muscle biopsies and/or animal models for diverse neuromuscular pathologies, including muscular dystrophies, neurodegenerative disorders, muscle denervation, and muscle overuse. In this context, CCN2/CTGF is deeply involved in extracellular matrix (ECM) modulation, acting as a strong pro-fibrotic factor that promotes excessive ECM accumulation. Reducing CCN2/CTGF levels or biological activity in pathological conditions can decrease fibrosis, improve muscle architecture and function. In this work, we summarize information about the role of CCN2/CTGF in fibrosis associated with neuromuscular pathologies and the mechanisms and signaling pathways that regulate their expression in skeletal muscle.

## Introduction

Fibrosis results from the excessive accumulation of extracellular matrix (ECM) proteins, such as collagens, fibronectin, and proteoglycans, in a scar-like tissue affecting several organs [1,2]. Skeletal muscle fibrosis can affect muscle function in different ways. The ECM stores and transmits forces from the muscle fibers to tendons and bones [4]. The continuity between the different ECM structures found in the skeletal muscle, the endomysium, perimysium, epimysium, and tendons, is essential for force generation. Therefore, changes in composition, organization, and quality of the ECM directly affect the force generated by the muscle. Under fibrotic conditions, in chronic diseases, the ECM excess also extends in the space previously occupied by contractile muscle fibers. Then reduced muscle force might result from decreased muscle fiber mass/number, concomitant to the increased ECM stiffness. Furthermore, ECM composition, and fibrosis, can mechanically affect not only contractile cells but also other cells present at the skeletal muscle such as fibroblast, vascular endothelial cells, immune cells, etc., modifying the concentration and availability of several growth factors and signaling molecules, greatly changing the tissue environment [[3], [4], [5],^12^]. On the other hand, fibrotic tissue becomes a physical barrier, a rigid wall, to diverse processes necessary for normal muscle function, such as neovascularization and reinnervation. Neovascularization is needed to reestablish proper oxygen and nutrient availability and is limited in fibrotic skeletal muscle [6,7]. Myoblast migration into damaged muscle can also be inhibited by the fibrotic wall, affecting muscle regeneration. This explains that diminishing fibrosis helps to more effective cell therapy in dystrophic muscle [[7], [8], [9]]. Also, the fibrotic skeletal muscle contains factors known to inhibit myoblast differentiation, and therefore, contribute to reduced muscle regeneration, affecting skeletal muscle function. For example, matricellular proteins such as Transforming growth factor β (TGF-β) and Cellular communication network 2/Connective tissue growth factor (CCN2/CTGF) have been shown to have an inhibitory effect on myoblast differentiation at different levels [[10], [11], [12], [13]]. Skeletal muscle fibrosis is associated with activation of transforming growth factor-beta (TGF-β), the best studied pro-fibrotic route in many tissues, including the skeletal muscle [14,15]. Furthermore, CCN2/CTGF role on tissue fibrosis has been also well studied [16,17]. Here, we review what we know about the role of CCN2/CTGF in skeletal muscle in neuromuscular pathologies and the mechanisms regulating its fibrotic activity.

## The matricellular protein CCN2/CTGF

Cellular communication network 2 or Connective tissue growth factor (CCN2/CTGF) is a matricellular protein involved in various critical biological processes, especially during embryonic development, where it is highly expressed. In adulthood, the expression of CCN2/CTGF is minimal in most tissues, but its levels increase in damaged tissues and various pathological conditions [[16], [17], [18]]. This protein is a member of the CCN family, characterized by the presence of four very well conserved modules (I to IV) [19,20]. Following the II structural domain, there is a non-conserved, unstructured “hinge” region of variable length that connects with the III domain. The cleavage of the CCN2/CTGF hinge region by proteinases releases the C-terminal fragment (III and IV domains), which retains at least some of the biological activities [21]. CCN2/CTGF has no canonical receptors but interacts with integrins, lipoprotein-related receptors, and tyrosine kinase receptors, stimulating signal transduction [16,[22], [23], [24]]. It can also interact with extracellular matrix (ECM) components such as heparan and sulfate proteoglycans [25], decorin [26], and fibronectin [27]. It has been suggested that these interactions with the ECM might compete with other factors, changing their local concentration and availability, and modulate matrix binding sites with consequences in cell motility and adhesion [17]. CCN2/CTGF has also been reported to interact with a variety of cytokines to alter their signaling activities [16]. For example, binding of CCN2/CTGF to TGF-β increases its binding to TGF-β receptors, stimulating its signaling. On the contrary, CCN2/CTGF interaction with bone morphogenetic protein (BMP) or vascular endothelial growth factor (VEGF) prevents binding to their receptors [28,29]. All these interactions can modify cell phenotype and occur separately or concurrently, having differential consequences depending on cell type and environment.

CCN2/CTGF abundance is elevated in human muscle biopsies and/or animal models for diverse neuromuscular diseases, including muscular dystrophies of different etiologies [8,[30], [31], [32]], neurodegenerative disorders [31,[33], [34], [35]], and muscle denervation [[36], [37], [38]]. Its role in the fibrotic response observed in several chronic diseases has been well documented [16,20,[39], [40], [41], [42]]. Our own studies and those performed by other groups showed that CTGF/CCN2 is involved in modulating skeletal muscle ECM. CCN2/CTGF is a robust pro-fibrotic factor that is upregulated in fibrotic areas, characterized by excessive accumulation of ECM proteins, and in necrotic/regenerative foci in damaged muscle [43,44]. Transient overexpression of CCN2/CTGF from an adenovirus injected into healthy skeletal muscle can trigger a transient fibrotic response and reduce muscle function [45]. Conversely, reduction of its levels under pathological conditions can decrease fibrosis and improve skeletal muscle performance [8,34,38].

## CCN2/CTGF in muscular dystrophies

CCN2/CTGF mRNA and protein levels are elevated in muscular dystrophies of different etiology. High levels of CCN2/CTGF has been reported in skeletal muscle biopsies from Duchenne muscular dystrophy (DMD), Becker muscular dystrophy, and Fukuyama-type congenital muscular dystrophy patients [31,46], and in samples from murine models for DMD and Limb-Girdle muscular dystrophy (LGMD) [30,32,47]. CCN2/CTGF has been more studied in DMD than in any other muscular dystrophy. DMD patients have higher CCN2/CTGF protein levels in skeletal muscle than control individuals, and the severity of the disease shows a direct correlation with CCN2/CTGF expression levels [48]. In the *mdx* mouse model of DMD, CTGF localizes in skeletal muscle necrotic foci isolated by laser capture microdissection. Analysis of these necrotic foci samples shows that CCN2/CTGF appears to be involved in the fibrotic and inflammatory responses. However, it does not participate in decreasing regeneration, since fibrosis and inflammation markers are reduced in muscle from *mdx* mice that express lower levels of CCN2/CTGF but satellite cells, the progenitors of muscle fibers, remain unchanged [44]. Therefore, CCN2/CTGF has been suggested as a critical therapeutic target for fighting the fibrotic response in muscle diseases [17].

**Fig. 1:**
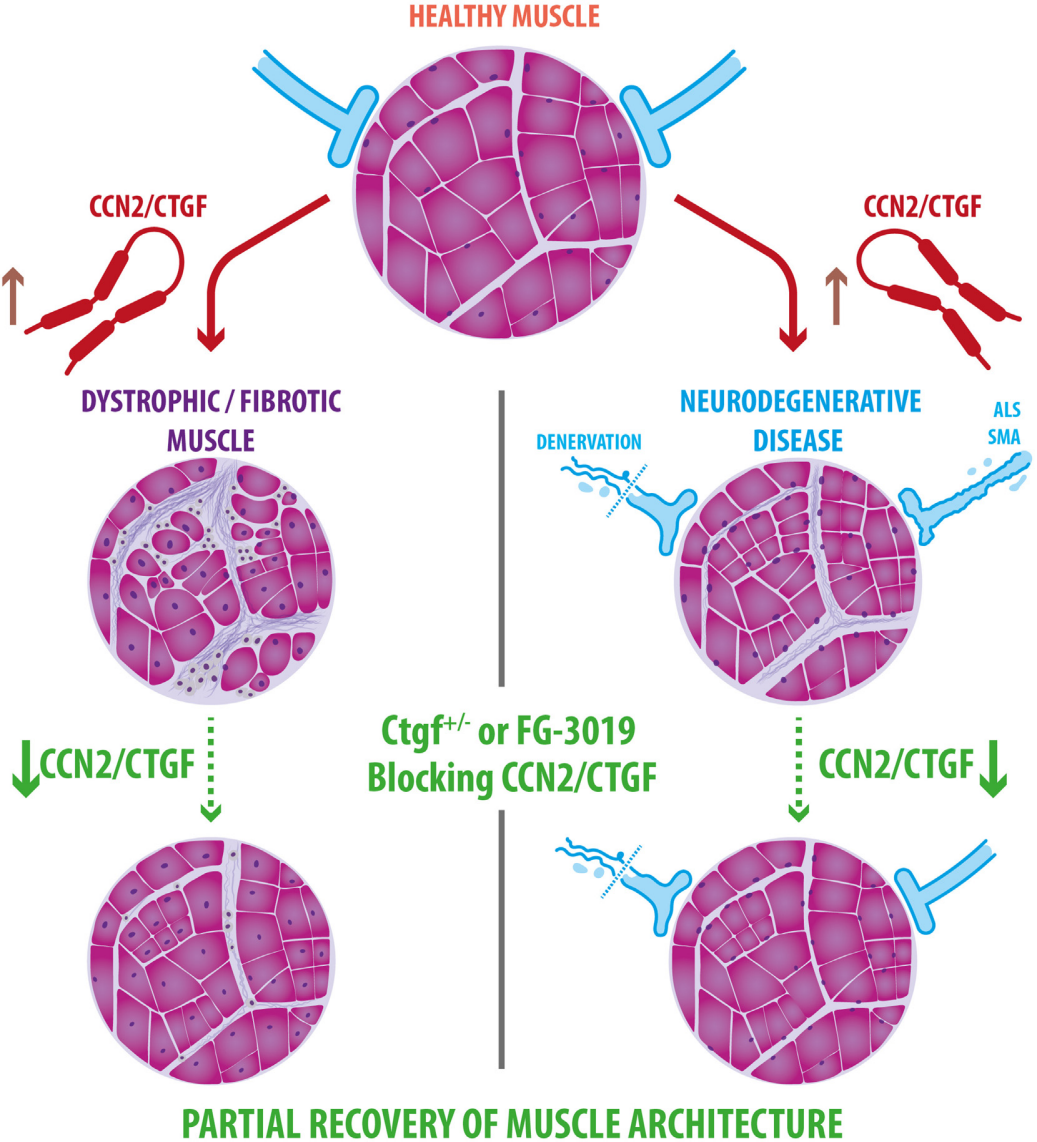
CCN2/CTGF is associated with neuromuscular diseases. Normal skeletal muscle presents homogenous sized myofibers, surrounded by normal levels of ECM. In muscular dystrophies and muscle overuse (schematized on the left), there is a heterogeneity in the size of muscle fibers, an augmented number of FAPs and myofibroblasts, and excessive accumulation of ECM proteins. In neurodegenerative diseases and denervation, death or traumatic section of motoneurons interrupt the communication of the nervous system with skeletal muscle (schematized on the right). Myofibers atrophy, there is an increment of FAPs, and increased accumulation of ECM. The fibrotic phenotype in any of these pathologies is associated with increased levels of CCN2/CTGF, which is a strong pro-fibrotic factor. Consequently, the partial recovery of normal muscle can be achieved through strategies that decrease CCN2/CTGF levels or activity, which in turn lead to the reduction of fibrosis and improvement of skeletal muscle architecture and function, as has been shown in models for DMD, ALS, muscle overuse, and denervation.

Therapeutic strategies that can minimize fibrosis are important since they can improve vascularization and facilitate cell therapy in dystrophic muscle [7,8]. Given its pro-fibrotic action, the blockage of CCN2/CTGF is an attractive approach. The genetic reduction of CTGF levels (Ctgf^+/−^) in the *mdx* mouse model increases tetanic muscle force, decreases muscle damage, apoptosis, and reduces the accumulation of ECM proteins such as fibronectin and collagen [8] (Fig. 1). Furthermore, the systemic treatment of *mdx* mice with pamrevlumab (FG-3019, FibroGen), a specific antibody able to block CCN2/CTGF biological activity [17,49], has similar effects as reducing the genetic load of Ctgf: it reduces fibrosis levels, improves tetanic muscle force, and facilitates cell therapy [8]. Regular exercise can exacerbate CCN2/CTGF expression and fibrosis in dystrophic skeletal muscles due to the fragility of the myofibers [46]. In normal muscle, intense training, or work-related overuse can lead to musculoskeletal disorders characterized by inflammation, muscle degeneration, and fibrosis [50,51]. The blockage of CCN2/CTGF also decreases the fibrotic response in an established and clinically relevant rat model of upper extremity overuse injury that induces muscle fibrosis [52].

These results, obtained in DMD animal models and overuse-induced fibrosis, are encouraging for the treatment of persistent skeletal muscle fibrosis with pamrevlumab. Notably, pamrevlumab has completed an open-label, single-arm Phase 2 clinical trial in non-ambulatory DMD patients where each subject received pamrevlumab for up to 156 weeks (NCT02606136). Preliminary results from this clinical showed that pamrevlumab helps to slow the decline, or even improve, lung and heart function and skeletal muscle strength in non-ambulatory boys and young men with DMD. Overall, pamrevlumab was well tolerated and showed promising results in slowing disease progression across all the studied parameters when compared with published data [53]. Currently, a newly initiated phase 3 clinical trial LELANTOS (NCT04371666), is enrolling non-ambulatory DMD patients on a stable dose of corticosteroids, will be randomized to receive with either pamrevlumab or placebo in combination with corticosteroids, every two weeks for up to 52 weeks.

## CCN2/CTGF in muscle denervation

After muscle denervation, the loss of communication with motor neurons leads to loss of voluntary contraction, rapid loss of muscle mass driven by activation of catabolic pathways, and eventually fiber degeneration. Fibrosis was considered a characteristic of very long-term denervated muscles [54]. Nevertheless, we showed that the activation of pro-fibrotic pathways and ECM accumulation occurs very early after muscle denervation. Accumulation of collagen I and fibronectin can be observed as soon as two days after sciatic nerve section in wild-type mice [38]. It was recently shown that stretching denervated muscles can decrease denervation-associated fibrosis [55], suggesting that the fibrotic process is highly dependent on the lack of muscle contraction and not only on the loss of neural input, which is also supported by the fact that disused skeletal muscle can also present fibrotic processes [56]. Ccn2/Ctgf gene expression was shown to be increased, together with the expression of other genes, in response to skeletal muscle denervation, although no biological significance was suggested for that finding [37]. Later, we showed that CCN2/CTGF mRNA and protein levels increase very quickly after denervation of skeletal muscle [38].

Reducing CCN2/CTGF levels using Ctgf^+/−^ mice or CTGF blocking antibodies (pamrevlumab/FG-3019) resulted in improved muscle architecture and decreased denervation-induced fibrosis [38] (Fig. 1). Interestingly, CCN2/CTGF upregulation occurs even before TGF-β mediated signaling, a pathway known to induce CCN2/CTGF expression, is fully activated. In that study, we proposed that at least in the early stages (2–4 days after denervation), CCN2/CTGF overexpression is independent of canonical TGF-β signaling, suggesting that other signals might induce CCN2/CTGF expression in denervated skeletal muscle [38]. Below, we discuss different signaling pathways involved in CCN2/CTGF upregulation.

## CCN2/CTGF in neurodegenerative diseases

In neurodegenerative diseases such as Amyotrophic Lateral Sclerosis (ALS) and Spinal Muscular Atrophy (SMA), motor neuron degeneration leads to loss of neuromuscular communication, as occurs in muscle denervation. The skeletal muscle progressively loses voluntary movement and atrophies. The mechanisms affecting skeletal muscle in motor neuron degenerative diseases are probably the sum of primary pathological features of the disease (e.g., accumulation of mutant SOD1 in some ALS patients) and secondary features due to progressive denervation [[57], [58], [59]]. CCN2/CTGF has been suggested to have a role in the progression of neuromuscular and neurodegenerative pathologies [42]. CCN2/CTGF overexpression was found in biopsies from SMA patients [31] and in the spinal cord of sporadic and familial ALS patients, in astrocytes and motor neurons [35]. The skeletal muscle from the transgenic mice expressing hSOD1^G93A^, a murine model for ALS, shows increased atrophy and fibrosis, evidenced by the accumulation of ECM proteins such as fibronectin and collagens, increased TGF-β signaling, and overexpression of CCN2/CTGF [33,34]. In this ALS model, treatment with the CCN2/CTGF blocking antibody FG-3019 ameliorates ALS-related pathology, improving the ability of mice to move around and enhancing muscle performance [34]. Interestingly, the effects of blocking CCN2/CTGF in neuromuscular diseases are not limited to reduced fibrosis and improved skeletal muscle pathology but can also improve or protect neuronal inputs (Fig. 1). Blocking CCN2/CTGF in the ALS mouse model decreases axon degeneration and preserves the structure of the neuromuscular junction (NMJ) [34]; while in the rat model of muscle overuse, it improves mononeuropathy, decreasing neural fibrosis and sensory decline [60]. The roles for CCN2/CTGF in the nervous system might emerge as new therapeutic targets not only for the treatment of muscular diseases but also for treating degenerative pathologies of the central nervous system [42,[61], [62], [63]].

## Mechanisms involving CCN2/CTGF upregulation in diseased skeletal muscle

**Fig. 2:**
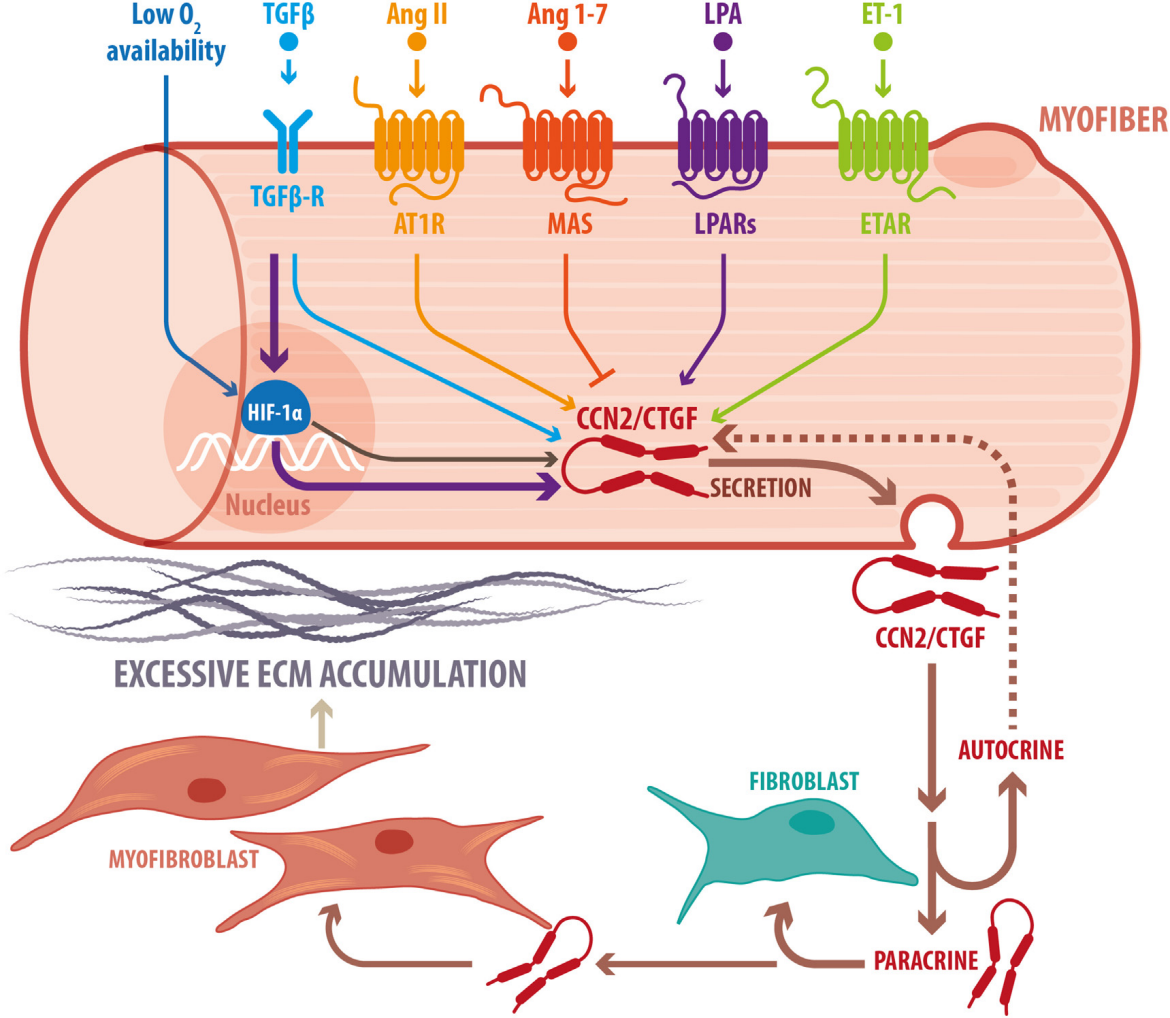
Signaling pathways involved in CCN2/CTGF upregulation in the skeletal muscle. Several signaling factors that are concomitantly increased in damaged skeletal muscle, can contribute to the upregulation of CCN2/CTGF. Because the role of CCN2/CTGF on skeletal muscle fibrosis has been mainly linked to the CCN2/CTGF protein produced in myofibers, these pathways are schematized in myofibers, although they can also function in other cell types. The signaling pathways that upregulate CCN2/CTGF include TGF-β signaling and hypoxia response through HIF-1α, which together act synergistically on myofibers. Also, signaling through the activation of specific G protein-coupled receptors by Endothelin-1, LPA, and the vasoactive RAS member Angiotensin-II has been shown to be involved in the upregulation of CCN2/CTGF. On the other hand, activation of the alternative branch of RAS by Angiotensin-(1-7) has protective effects on skeletal muscle and reduces CCN2/CTGF. Secretion of CCN2/CTGF from muscle fibers can now act as an autocrine signal on the same muscle fibers or as a paracrine signal on surrounding cells, such as immune cells, endothelial cells in blood vessels, and FAPs, which differentiate to myofibroblasts, increasing their fibrotic potential. No receptors for CCN2/CTGF are shown because no canonical receptors have been described to date. However, CCN2/CTGF can interact with integrins, lipoprotein related proteins, tyrosine kinase receptors, and ECM components as well as a variety of cytokines.

CCN2/CTGF can be upregulated by different signals that are induced concomitantly in damaged or diseased skeletal muscle. Below is a summary of some of these signals and how this regulation of CCN2/CTGF is achieved in the different cell types that form the skeletal muscle (Fig. 2).

Skeletal muscle fibrosis is associated with activation of transforming growth factor-beta (TGF-β), the best studied pro-fibrotic route in many tissues, including the skeletal muscle [14,15]. The canonical or Smad-dependent TGF-β signaling leads to the expression of target genes, such as collagens, proteoglycans, and fibronectin, which contribute to the development of fibrosis in different skeletal muscle pathologies [28,33,38,64,66]. CCN2/CTGF interaction with TGF-β increases binding to cell surface receptors favoring TGF-β signaling [28]. Thus, as Ccn2/Ctgf is also a target gene for TGF-β signaling, it generates positive feedback that contributes to fibrosis development when both factors remain elevated, as in chronic damage or pathology [67]. The fibrotic actions of TGF-β signaling can target different cells, including myofibers, fibroblasts, and fibroadipogenic progenitors (FAPs), which differentiate to myofibroblasts with increased fibrotic potential [68,69]. Furthermore, CCN2/CTGF also induces diverse cells' transdifferentiation into fibroblast and myofibroblasts, increasing the cells with high fibrotic potential [65,[70], [71], [72], [73]].

Damaged or diseased skeletal muscle presents stabilization and nuclear localization of hypoxia-inducible factor 1α (HIF-1α), indicating that the hypoxia response is active [74]. Chronic hypoxia by itself might compromise muscle function and has been linked to muscle atrophy and reduced force [75]. Hypoxia and the hypoxia-inducible factor 1α (HIF-1α) have been involved in establishing fibrosis in different cell types, including skeletal muscle [6]. Furthermore, sustained pharmacological stabilization of HIF-1α can increase CCN2/CTGF levels and ECM accumulation in skeletal muscle [6]. We have shown that hypoxia can act together with TGF-β signaling to upregulate CCN2/CTGF synergistically. Interestingly, this synergic upregulation of CTGF occurs only on differentiated myotubes and myofibers [74]. Supporting the importance of myofibers on the role of CCN2/CTGF in skeletal muscle, Petrosino et al. showed that selective ablation of CCN2/CTGF in myofibers, but not in fibroblasts, can reduce fibrosis and improve muscle function in dystrophic mice [76]. These observations suggest that under pathological conditions, the hypoxic environment and increased TGF-β signaling induce myofibers to secrete high amounts of CCN2/CTGF. In addition to acting in an autocrine manner to affect myofibers, secreted CCN2/CTGF can also act in a paracrine manner on neighbor cells, such as FAPs, stimulating the excessive expression of ECM proteins. Furthermore, CCN2/CTGF inhibits VEGF angiogenic action, which contributes to maintaining the low oxygen supply and hypoxic condition, generating another positive feedback for CCN2/CTGF upregulation and fibrosis establishment in skeletal muscle pathology [29].

Proteins known to act on the cardiovascular system have also been linked to the modulation of CCN2/CTGF. Receptors for endothelin-1, a vasoconstrictor, can induce CCN2/CTGF expression and fibrotic response in different tissues [[78], [79], [80], [81]]. Angiotensin II (Ang-II), a member of the renin-angiotensin system (RAS), can induce CCN2/CTGF [77][[82], [83], [84]], while inhibition of the Angiotensin-Converting Enzyme (ACE), which produces Ang-II, decreases CCN2/CTGF expression in dystrophic mice, reducing fibrosis and improving muscle force [47]. Angiotensin 1-7 (Ang-1-7), a member of the anti-fibrotic branch of RAS [85], reduces CCN2/CTGF expression [86] concomitantly with a decrease in fibrosis and improvement of skeletal muscle physiology in the *mdx* mouse model [87].

The bioactive lipid lysophosphatidic acid (LPA) has been involved in the development of fibrosis in different tissues [[88], [89], [90], [91]]. LPA is produced from extracellular lysophospholipids by the enzyme autotaxin and it is also synthesized by phospholipases from membrane glycerophospholipids [92,93]. LPA signals through six receptors (LPA_1_-LPA_6_), which are cell-surface G protein-coupled receptors (GPCRs) [94]. In skeletal muscle cells, LPA can induce CCN2/CTGF overexpression [10,95,96]. Experimental evidence shows that LPA is involved in inflammation and fibrotic reactions associated with neuromuscular diseases [97]. We have found that CCN2/CTGF expression in skeletal muscle in response to denervation is mediated by LPA_1_/LPA_3_ (Cordova-Casanova et al., unpublished results). The mechanisms that control the expression of CCN2/CTGF in response to LPA are complex, requiring GPCRs [98], RhoA [99], TGF-β and JNK [95] signaling, cytoskeletal organization [100,101], and the hippo-YAP pathway [102]. Similar observations have been made in peritoneal mesothelial cells, where LPA induces CCN2/CTGF, which is required for fibroblast cell proliferation and peritoneal fibrosis [103,104]. Interestingly, a new regulatory pathway involving a loop between CCN2/CTGF and YAP has been proposed for angiogenesis [105]. Further investigation on this area in the context of skeletal muscle fibrosis is required.

## Concluding remarks

CCN2/CTGF is a strong pro-fibrotic factor that has an important role in establishing the skeletal muscle scar-like deposition of connective tissue, characteristic of many neuromuscular disorders of diverse etiology. The best studied inducer of CCN2/CTGF is TGF-β signaling, but there are other concomitant signaling pathways in damaged skeletal muscle that have been shown to induce its expression. Although there is evidence showing that myofibers might be the primary producers of CCN2/CTGF, which can then act as an autocrine and paracrine signal, we need further investigation concerning the cell-specific mechanisms of CCN2/CTGF action in skeletal muscle diseases. Today, there are candidate therapeutic approaches that reduce CCN2/CTGF levels or activity, which improve muscle function and increase the quality of life of patients suffering from neuromuscular diseases.

## Declaration of competing interest

KEL is an employee and shareholder of FibroGen, Inc. The other authors have no conflicts of interest.
